# Sweeteners at the Water–Soil Interface and in Soil Matrices: A Brief Review of Ecotoxicological Impacts

**DOI:** 10.3390/ijms27219674

**Published:** 2026-10-29

**Authors:** Volodymyr V. Tkach, Maria do Rosário Melo da Costa, Tetiana V. Morozova, João Ricardo Sousa, Yana G. Ivanushko, José Inácio Ferrão de Paiva Martins, Isabel Gaivão

**Affiliations:** 1 Animal and Veterinary Research Center (CECAV), Associate Laboratory for Animal and Veterinary Sciences (AL4AnimalS), University of Trás-os-Montes and Alto Douro (UTAD), 5000-801 Vila Real, Portugal; 2 Centre for the Research and Technology of Agro-Environmental and Biological Sciences (CITAB), University of Trás-os-Montes and Alto Douro (UTAD), 5000-801 Vila Real, Portugal; jricardo@utad.pt; 3 GeoBioTec Research Centre, University of Aveiro, 3810-193 Aveiro, Portugal; rosario.costa@utad.pt; 4 Geology Department, University of Trás-os-Montes and Alto Douro (UTAD), 5000-801 Vila Real, Portugal; 5 State Scientific Institution “Institute of Ecological Restoration and Development of Ukraine”, Environment Remediation Section, Mytropolite Vasyl Lypkivsky Str. 35, 01001 Kyiv, Ukraine; tetiana.morozova@ukr.net; 6 Disaster and Military Medicine Department, Bukovinian State Medical University, Teatralna Sq. 9, 58001 Chernivtsi, Ukraine; yana_iv@ukr.net; 7 Engineering Faculty, University of Porto, Rua Dr. Roberto Frias, s/n, 4200-065 Porto, Portugal; jipm@fe.up.pt

**Keywords:** soil ecology, soil–water interface, sugar substitutes, ecotoxicity, genotoxicity, environmental impact, environmental fate

## Abstract

Sweeteners have become emerging contaminants in terrestrial and aquatic environments due to their extensive use in food, pharmaceutical, alternative smoking, and personal care products. They reach terrestrial ecosystems from both atmospheric and aquatic ecosystems, making the water–soil interface a critical zone governing their transport, persistence, and environmental fate, which remains mostly unknown, thus creating a gap that needs to be filled. This review aims to summarize current knowledge on the occurrence, mobility, persistence, and ecotoxicological effects of artificial and natural sweeteners in soils and at the water–soil interface, with emphasis on sucralose, saccharin, acesulfame potassium, aspartame, cyclamate, and steviol glycosides. The review covers seventy-two full papers, published from 2004 to 2026, involving sweetener intake and occurrence and environmental fate in soil and at the water–soil interface. Sweeteners may persist in terrestrial and aquatic ecosystems, inducing, either individually or in combination, oxidative stress and influencing microbial communities and enzymatic activity at the soil–water interface, which affects invertebrates, plant roots, and the overall ecosystem. Their effects on soil biota are still poorly understood. These influences, as well as the effects of long-term persistence and differences in responses across soil types and climatic conditions, are mostly unknown. This review highlights the central role of the soil matrix and water–soil interface in understanding the environmental fate and ecological significance of sweeteners and identifies key gaps requiring further soil-specific research.

## 1. Introduction

Soil health is a fundamental component of terrestrial ecosystems and sustainable agricultural production, directly influencing nutrient cycling, water retention, biodiversity, and plant productivity [1,2,3,4], independently of season, climatic zone, and urbanization degree. Healthy soils support essential ecological functions and contribute to climate regulation, food safety, and environmental resilience. However, increasing anthropogenic pressure associated with industrialization, urbanization, and intensive agricultural practices has accelerated the input of numerous contaminants into soil environments, raising concerns regarding the long-term preservation of soil quality and ecosystem functioning [5,6,7].

Among the major threats to soil health is the continuous input of xenobiotics [8,9,10], originating from multiple sources, including industrial effluents, pharmaceutical residues, pesticides, personal care products, sewage sludge, and wastewater reuse. Once in soils, xenobiotics may alter physicochemical properties, disrupt microbial communities, and affect biogeochemical cycles, which are essential for ecosystem stability [11,12].

The environmental behavior of xenobiotics in soils and at the soil–water interface depends on several factors, including chemical structure, persistence, mobility, bioavailability, and interactions with soil components. Some compounds are quickly degraded, whereas others exhibit significant environmental persistence, thereby being capable of accumulating over time [13,14,15]. Their effects may include changes in soil enzymatic activity, disruption of microbial diversity, phytotoxicity, genotoxicity, DNA alterations, and adverse impacts on soil fauna. Consequently, the growing accumulation of emerging contaminants in terrestrial ecosystems has become an important topic in environmental and soil sciences [16,17,18].

Artificial sweeteners have recently attracted attention as emerging xenobiotic contaminants due to their widespread consumption and increasing environmental occurrence [19,20,21]. These compounds are extensively used in food products, beverages, alternative tobacco products, pharmaceuticals, and personal care formulations as sugar substitutes with low or negligible caloric value. Other important and growing sources of artificial sweeteners are tobacco-related products, particularly electronic cigarettes and oral nicotine products, where they are added to improve flavor perception and mask the bitterness of nicotine. The increasing popularity of these products has further contributed to the overall consumption and environmental release of synthetic sweeteners.

As many artificial sweeteners are highly stable in conventional wastewater treatment sets, they penetrate aquatic and terrestrial environments, raising concerns about their persistence and potential ecological effects. Saccharin, cyclamate, acesulfame potassium, aspartame, and sucralose have been detected in aquatic and terrestrial environments worldwide [22,23,24,25,26], with sucralose being the most ubiquitous. Their continuous release into the environment has raised concerns regarding their persistence, transformation products, and potential ecotoxicological effects. These effects are described mostly for aquatic systems and, to a lesser extent, for the water–soil interface, but much less is known about their behavior in the soil itself, leading to possible unforeseen consequences for soil ecosystems [22,23].

Investigating the impacts of artificial sweeteners on soil health and the soil–water interface is of high importance, given the growing global consumption of these compounds and the expansion of wastewater reuse in agriculture [27,28]. Since soils act as both sinks and transformation media for contaminants, understanding the environmental behavior of sweeteners in the lithosphere is essential for evaluating ecological risks and maintaining sustainable soil management practices. Furthermore, the interaction of sweeteners with soils—including sorption; chemical stability; and impact on microorganisms, invertebrates, and organic matter—may influence nutrient cycling and ecosystem functioning.

The assessment of sweeteners in soils also contributes to the broader discussion of emerging contaminants and environmental monitoring [29,30,31,32]. While significant research efforts have focused on aquatic ecosystems, terrestrial environments remain comparatively understudied. As a result, important questions persist regarding the fate, transport, degradation pathways, and cumulative ecotoxicological effects of these compounds under different soil and climatic conditions [33,34,35,36].

Despite growing scientific interest in artificial sweeteners as environmental contaminants, available studies on their occurrence and ecotoxicological impacts in soils remain limited and fragmented. Existing research often focuses on aquatic systems, and terrestrial ecosystems receive comparatively less attention. Moreover, methodological differences among studies complicate direct comparisons and hinder the establishment of comprehensive environmental risk assessments, underscoring the need for further research on the environmental fate of sweeteners in soils. Therefore, reviewing and integrating current knowledge are necessary to identify major trends, research gaps, and future priorities in this emerging field [37,38,39,40].

The present review aims to summarize and critically evaluate the most recent scientific literature on the occurrence, persistence, environmental behavior, and ecotoxicological impacts of artificial sweeteners in soils and their interface with aquatic ecosystems, with emphasis on their potential implications for soil health and ecosystem functioning. Additionally, this narrative review seeks to identify existing knowledge gaps and highlight directions for future research concerning these emerging soil and water contaminants [37,38,39,40].

## 2. Materials and Methods

Articles for this review were selected using a structured and transparent methodology. A comprehensive literature search was conducted across major scientific databases, including Web of Science, Scopus, and PubMed, using targeted keywords related to the biological impact of natural and artificial sweeteners in soils and their environmental fate, ecotoxicity, genotoxicity, oxidative stress, and recycling. An example of the Boolean operators used in the literature search is as follows:

TITLE-ABS-KEY((sweetener* OR “sugar substitute*” OR sucralose OR saccharin OR aspartame OR acesulfame OR “acesulfame potassium” OR cyclamate OR “steviol glycoside*” OR stevia) AND (soil* OR terrestrial OR sediment*))

To identify studies related to sweeteners’ environmental fate in soil systems, the following were used:

TITLE-ABS-KEY((sweetener* OR “sugar substitute*” OR sucralose OR saccharin OR aspartame OR acesulfame OR “acesulfame potassium” OR cyclamate OR “steviol glycoside*” OR stevia) AND (soil* OR terrestrial) AND (ecotoxic* OR genotoxic* OR toxicity* OR “environmental fate” OR persistence OR mobility))

The sources were consulted seven times between 12 June and 25 August 2026 and included full papers, published between 2006 and the first half of 2026. The inclusion criteria focused on the relevance to the review topic (sweetener occurrence and environmental fate), methodological rigor, and publication in highly cited peer-reviewed journals. In order to ensure a thorough and up-to-date overview of the field, additional references were identified through citation tracking. Following the initial screening of titles and abstracts, full-text articles were evaluated to confirm their relevance and quality. Non-peer-reviewed manuscripts, including preprints and short conference papers, were excluded until peer review, necessary to ensure research quality, was completed. The detailed description of the study selection and the approach taken during it is described in Appendix A. 

**Figure 1:**
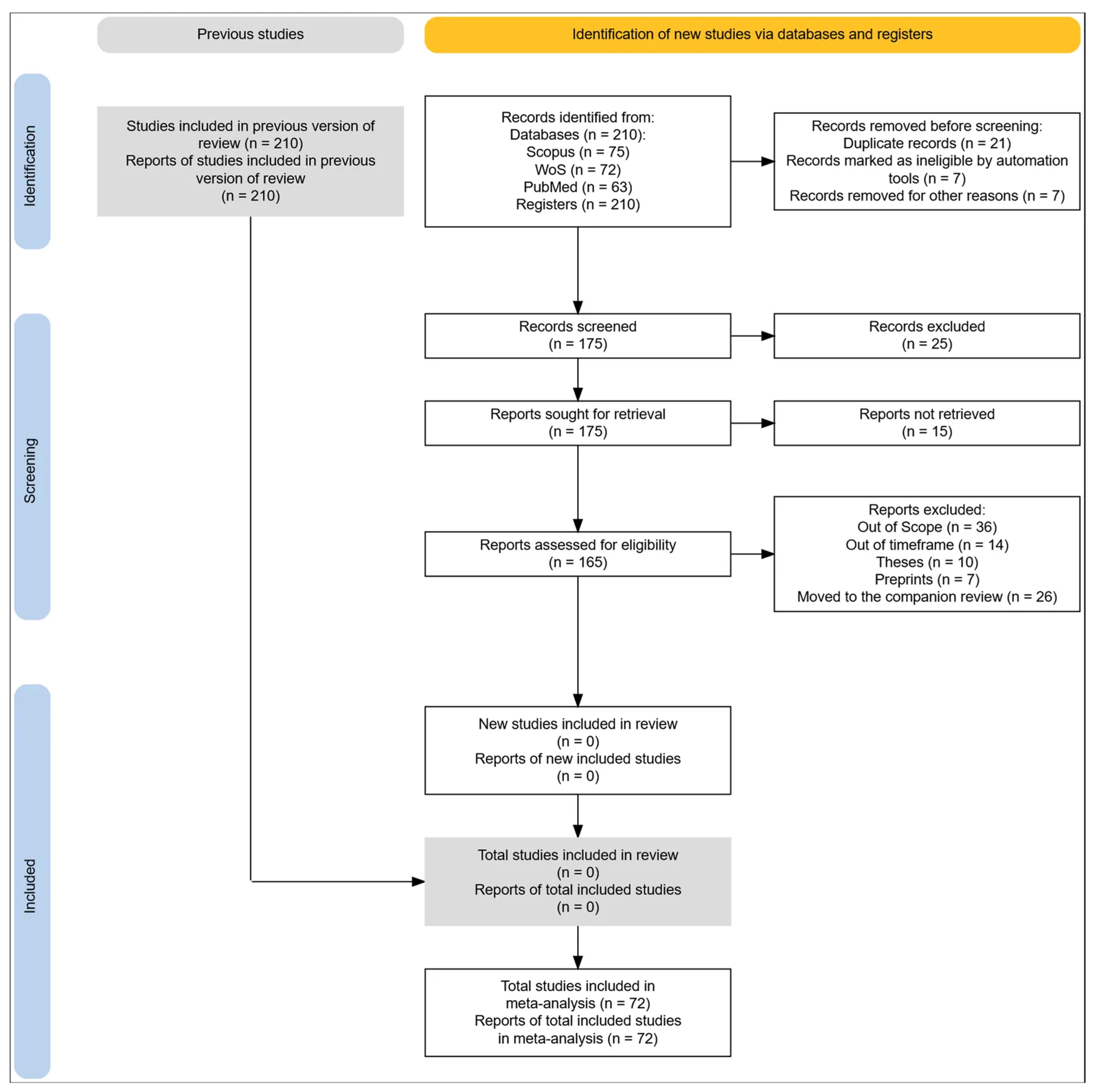
PRISMA flow diagram of the study selection process. Of the 210 initially identified sources, 98 studies remained after removal of duplicates, preprints, irrelevant studies, and theses. Of these, 26 were allocated to a companion review on sweetener determination in soils, leaving 72 studies for the present work.

This systematic approach ensured the inclusion of high-quality studies published over the last 20 years, providing valuable insights into current advances and ongoing challenges in detecting the presence of sweeteners, assessing their biological impact, and understanding their environmental fate in soils and at the soil–water interface. After analysis of the 210 initial sources, duplicates, preprints, irrelevant studies, and theses were removed, and 98 studies remained. Of these, 26 were allocated to a companion review focusing on the determination of sweetener concentrations in soils. Therefore, 72 articles were selected for this study, as shown in Figure 1.

A total of 72 studies describing the behavior and environmental fate of sweeteners in soils and at the soil–water interface were selected and examined in this review.

**Figure 2:**
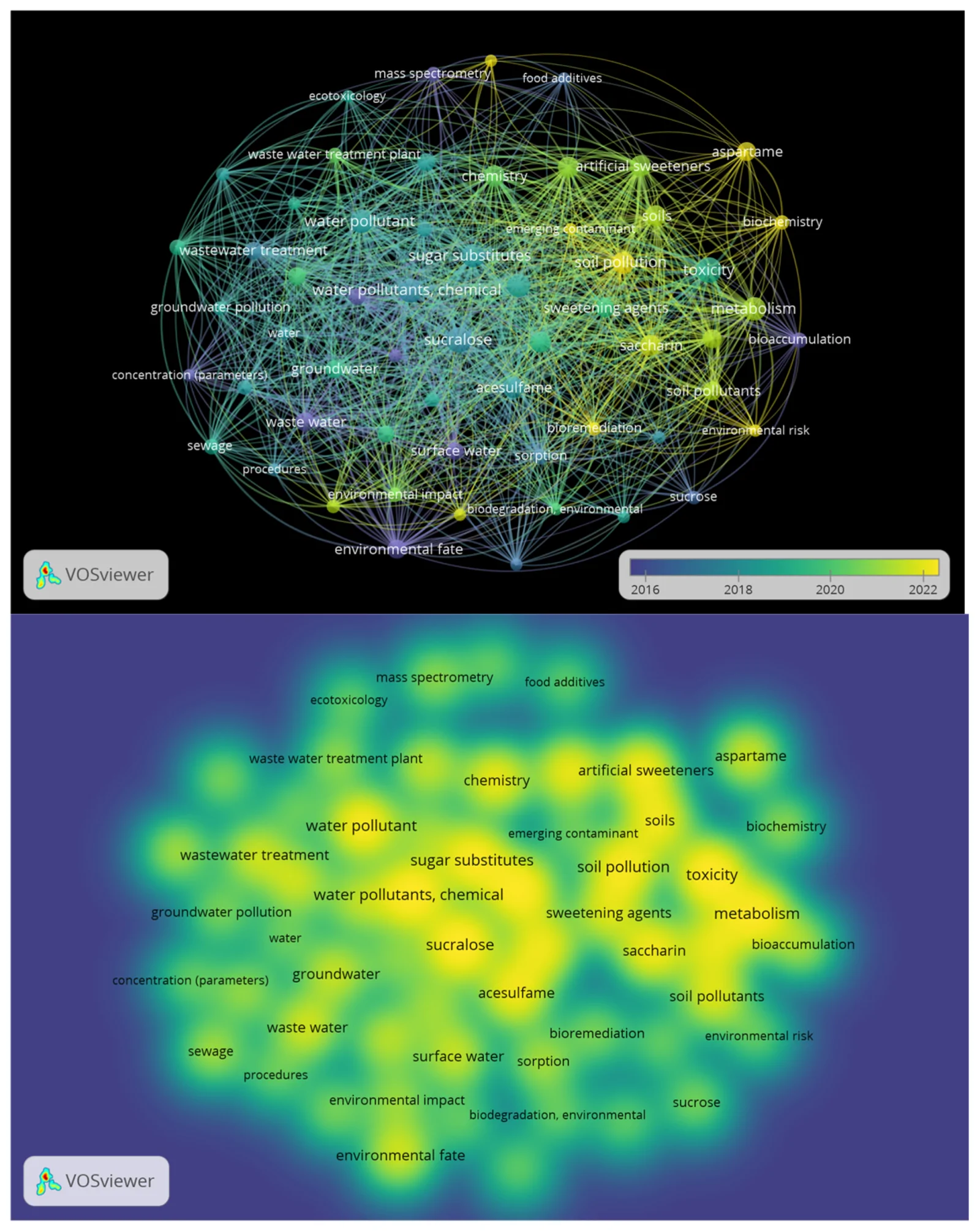
Keyword co-occurrence network overlay (upper) and density (lower) visualization. The colors in the network overlay diagram correspond to the years in which the keywords mostly co-occur, gradually shifting from 2016 (blue) to after 2022 (bright yellow). The nodes indicate the frequency of use of each keyword cited, whereas the thickness of the lines between them indicates the frequency of the keywords’ co-occurrence (the more frequently the keywords, their synonyms, and related words in the semantic field co-occur, the thicker the lines between them). In the keyword co-occurrence density visualization, a brighter color indicates more frequent co-occurrence, whereas green, cyan, and blue indicate less frequent co-occurrence. Both diagrams indicate that ecotoxicological investigations of each of the mentioned sweeteners became more intense in the 2020s (indicated by a brighter color in the network overlay visualization and wider areas in the density visualization) and that their environmental fate in soils is still poorly understood (indicated by blue, cyan, and green elements in the lower right part of the diagram).

In order to better analyze the obtained information, a keyword co-occurrence network overlay and density visualization was obtained using VOSviewer (Figure 2).

The keyword co-occurrence network, generated using VOSviewer 1.6.21, provides an overview of the main research themes related to sweeteners in terrestrial environments. The network analysis identified three major thematic clusters: The first cluster includes wastewater, groundwater, environmental fate, and water pollution, reflecting the occurrence and transport of sweeteners in aquatic systems. The second cluster includes soils, soil pollution, toxicity, metabolism, bioaccumulation, and artificial sweeteners, highlighting research focused on the environmental behavior and ecological effects of sweeteners in terrestrial and water–soil interface ecosystems. The third cluster includes analytical chemistry and mass spectrometry, representing the analytical methodologies used for the detection and characterization of these compounds.

The density visualization and keyword co-occurrence analysis revealed major research hotspots around “sugar substitutes,” “sucralose,” “aspartame,” “saccharin,” “artificial sweeteners,” “soil pollution,” “toxicity,” and “metabolism,” together with related terms in their semantic fields. These interconnected keywords indicate growing research interest in the environmental occurrence, fate, and ecotoxicological effects of artificial sweeteners, particularly sucralose, saccharin, and aspartame, with considerable emphasis on their analytical detection in environmental matrices. Despite this increasing attention, their fate in soils remains insufficiently understood, particularly regarding their long-term persistence, transport, transformation products, interactions with soil microorganisms and other contaminants, and ecological effects under realistic environmental conditions. These gaps highlight the need for further research on artificial sweeteners in terrestrial ecosystems.

## 3. Results

The results described in this review may be divided into two main thematic sections: The first examines their biological and ecotoxicological impacts on soil and wetland microbial communities, fauna, plants, and overall ecosystem health. The second analyzes their environmental fate, focusing on their persistence, mobility, degradation, and interactions with other soil constituents. Together, these sections provide an integrated overview of the occurrence and ecological significance of artificial sweeteners in terrestrial environments.

### 3.1. Sweeteners’ Biological Impact in Soil Chars and at Soil–Water Interface

Artificial sweeteners (ASs) have been extensively investigated for their effects on human and animal gut microbiota, including alterations in microbial composition, metabolic activity, and community structure [41,42,43]. Although soil and gastrointestinal ecosystems share diverse microbial communities and some functional processes, they differ substantially in physicochemical conditions, nutrient availability, microbial composition, and metabolic and exposure pathways [44,45]. Therefore, findings from gut microbiome studies may provide useful mechanistic hypotheses, but their application to soils requires independent, soil-specific investigation.

Evidence of the biological effects of ASs in terrestrial systems is still limited but increasingly indicates that these compounds are not always biologically inert. In *Folsomia candida*, environmentally relevant concentrations of sucralose, saccharin, cyclamate, and acesulfame altered reproduction, metabolic pathways, and gut microbiota, with some responses becoming more pronounced or changing direction following multigenerational exposure [46]. Similarly, acesulfame affected metabolism, reproduction, and gut microbial communities in *Eisenia fetida* [47]. In soil and wetland microbial communities, sucralose has been associated with reduced microbial activity and altered community composition, while its high persistence may favor chronic exposure [48]. Other studies suggest that ASs may interact with co-occurring contaminants and influence the dissemination of antibiotic resistance genes. Sucralose enhanced extracellular antibiotic resistance gene enrichment in triclosan-conditioned systems, while cyclamate combined with enrofloxacin promoted resistance gene abundance and transfer in soil–water systems [49,50,51,52]. These observations suggest that the ecological effects of ASs may depend not only on their individual toxicity but also on their interactions with other anthropogenic contaminants.

Experimental evidence remains scarce for soil organisms, however, while studies using *Allium cepa* have reported chromosomal abnormalities and micronucleus formation following exposure to sucralose, aspartame, and saccharin, including indications of synergistic effects in mixtures [53,54,55,56,57]. These findings support the need to consider ASs in combination with other contaminants rather than exclusively as individual compounds. The potential genotoxicity of ASs and their transformation products represents an additional concern. Compounds associated with sweetener metabolism or degradation, including cyclohexylamine and 6-acetylsucralose, have been suggested to possess biological activity relevant to genetic stability [58,59].

Plant studies further indicate that ASs may be taken up and transformed by crops. Aspartame and saccharin were detected in wheat tissues, together with several transformation products, some of which were predicted to have greater toxicity than the parent compounds [60]. Exposure of wheat and barley to mixtures of pharmaceuticals and ASs induced oxidative stress and impaired photosynthetic and non-enzymatic antioxidant responses [61]. Nevertheless, phytotoxicity and soil-to-plant transfer remain insufficiently investigated, representing an important research gap [62,63].

The environmental effects of ASs are also strongly influenced by their fate and mobility in soil. Sorption studies indicate that pH, ionic strength, cation composition, and soil organic carbon can substantially modify the retention of compounds such as acesulfame and saccharin, with reported differences in sorption coefficients of up to sixfold [64]. Consequently, exposure of soil biota depends not only on the intrinsic properties of the individual sweeteners but also on local soil characteristics and environmental conditions.

The available evidence suggests that ASs should not be regarded solely as passive tracers of anthropogenic contamination. They may interact with soil microbial communities, invertebrates, and plants, affecting microbial diversity and metabolism, reproduction, physiological and oxidative responses, genetic stability, and potentially the dissemination of antibiotic resistance genes [48,49,50]. However, direct evidence from soil systems remains limited, particularly regarding chronic exposure, multigenerational effects, transformation products, mixture interactions, and impacts on ecosystem functioning. Further soil-specific studies are therefore required to determine whether ASs constitute biologically relevant emerging contaminants rather than merely persistent indicators of wastewater-derived pollution. A summary of the reported biological and ecological effects is presented in Table 1.

At the same time, the currently available literature remains highly fragmented and unevenly distributed across research areas. Most investigations have focused on aquatic systems, wastewater treatment processes, or individual laboratory-scale exposure experiments, whereas long-term field studies of terrestrial ecosystems remain scarce. Furthermore, many studies evaluate single compounds under controlled conditions, despite the fact that artificial sweeteners are commonly found in complex environmental mixtures alongside pharmaceuticals, pesticides, antibiotics, heavy metals, and other emerging contaminants. The combined and potentially synergistic effects of these substances on soil biota, plant physiology, and microbial resistance mechanisms remain insufficiently understood. In particular, the persistence of compounds such as sucralose raises important questions regarding chronic exposure, accumulation, and multi-generational ecological consequences.

An additional limitation of the available literature is the relatively limited understanding of how soil physicochemical properties regulate the environmental behaviour of artificial sweeteners. Although several studies have investigated their occurrence and persistence, comparatively few have systematically evaluated the influence of soil type, mineralogy, organic matter content, clay composition, cation exchange capacity, pH, and redox conditions on sorption, mobility, biodegradation, and transformation processes. Consequently, the environmental fate of sweeteners cannot yet be generalized across different terrestrial ecosystems. Furthermore, available data on sorption coefficients (K_d_), partitioning behaviour, and degradation kinetics and (eco)toxicological assessments of the resulting products, which are different, depending on soil type and environmental conditions, remain scarce for many compounds (and sweeteners are among such compounds), and often show considerable variability due to differences in experimental design and soil characteristics. Establishing standardized methodologies for evaluating these parameters under environmentally realistic conditions, including extremum exposures, would considerably improve the comparability of future studies and provide a stronger mechanistic basis for predicting the transport, persistence, bioavailability, and ecological risks of artificial sweeteners in agricultural and natural soils.

Future research should therefore prioritize integrated and interdisciplinary approaches capable of linking analytical chemistry, soil science, microbiology, ecotoxicology, and plant physiology. Greater attention should be devoted to the molecular mechanisms underlying sweetener-induced biological effects, including oxidative stress, metabolic disruption, changes in gene expression, and alterations in the microbiome. In addition, more environmentally realistic exposure scenarios are needed to assess the long-term ecological significance of artificial sweeteners in agricultural and natural soils in order to support sustainable soil management and protect the integrity of terrestrial ecosystems under increasing anthropogenic pressure. The environmental impact of these sweeteners in soils and at the lithosphere–hydrosphere interface is briefly reviewed in Section 3.2.

### 3.2. Sweeteners’ Environmental Fate in Soils and at Soil–Water Interface

#### 3.2.1. Soil Constituents and Physicochemical Controls on Sweetener Bioavailability

The environmental fate of sweeteners in soils depends not only on their molecular properties and concentration but also on the soil characteristics, including organic matter, clay mineralogy, pH, K_d_, redox conditions, texture, moisture, and microbial activity. Following their introduction via wastewater, sludge, manure, or runoff, these compounds enter a reactive, multiphase system in which solid-phase interactions regulate their partitioning, mobility, bioavailability, and transformation, affecting leaching, rhizosphere retention, microbial accessibility, and protection within organo-mineral domains. Although polar, water-soluble sweeteners such as acesulfame, saccharin, cyclamate, and sucralose are frequently detected in wastewater-impacted environments, their persistence varies substantially: cyclamate and saccharin are generally more labile, whereas acesulfame and particularly sucralose tend to be more persistent, with reported half-lives ranging from weeks to months and even years depending on the soil properties and environmental conditions.

Soil organic matter is a key regulator of bioavailability, with its chemical composition, aromaticity, polarity, humification, and physical organization influencing retention through hydrogen bonding, van der Waals forces, dipole interactions, hydrophobic partitioning, and cation bridging [65,66,67,68,69,70]. Sorption to organic matter can reduce the freely dissolved fraction, decreasing immediate leaching and acute exposure, but may also prolong environmental residence time by protecting contaminants from rapid biodegradation or creating slow desorption pools—relevant for chronic exposure and contaminant “aging,” where molecules diffuse into micro/nanopores and show reduced extractability over time.

The chemical diversity of sweeteners requires compound-specific interpretation: saccharin and acesulfame are often anionic, sucralose is neutral but highly polar and chlorinated, cyclamate is ionizable, and aspartame and steviol glycosides have distinct functional groups. Affinity for soil organic matter, mineral surfaces, and pore water thus cannot be generalized; for example, sucralose may exhibit low sorption under some conditions, with persistence driven more by degradation resistance and continuous input than strong retention.

**Figure 3:**
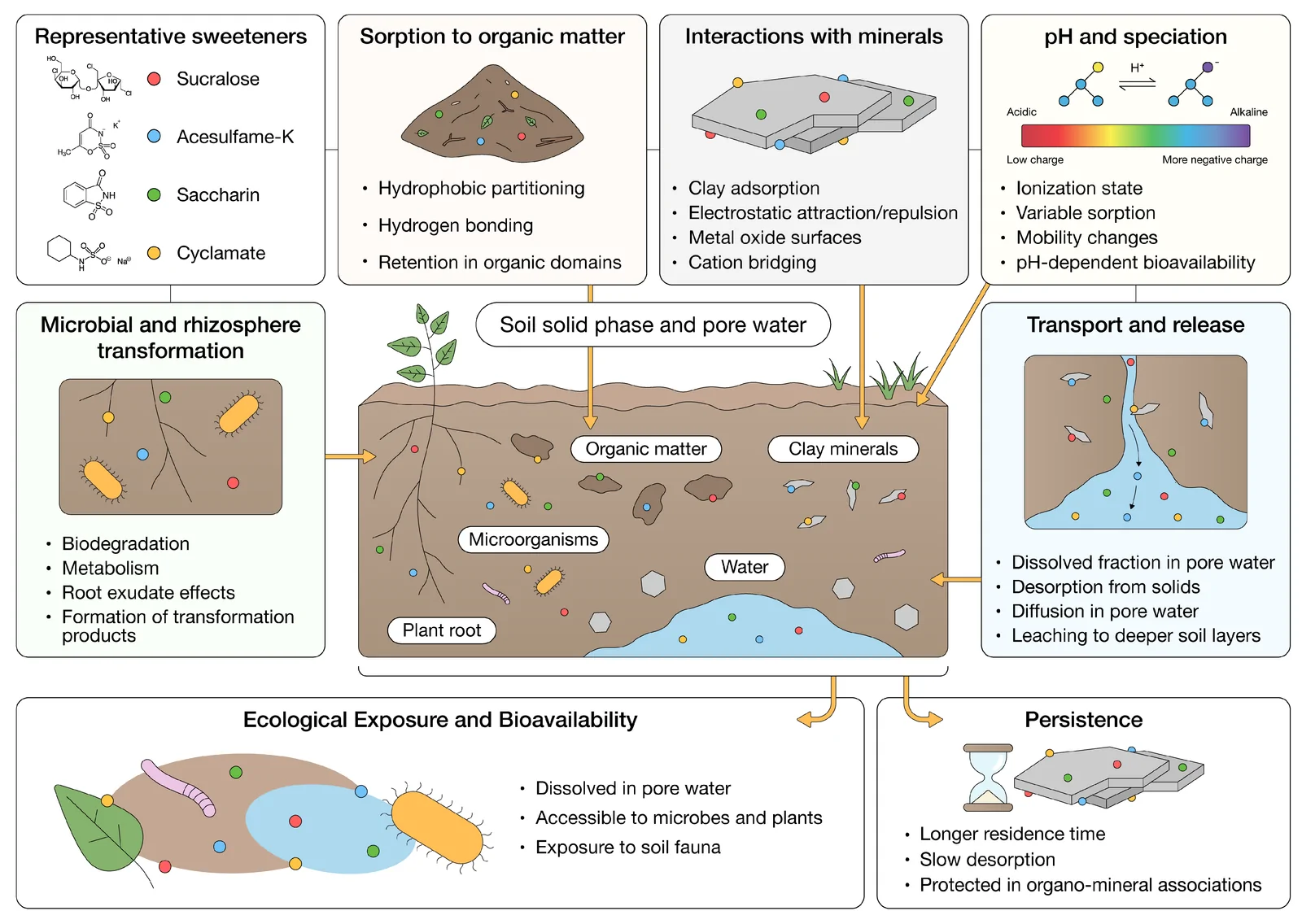
Soil solid-phase controls on sweetener transformation and bioavailability: conceptual framework for sorption, speciation, transformation, and ecological exposure of artificial sweeteners in soils.

The conceptual framework presented in Figure 3 illustrates how soil properties govern sorption, transformation, mobility, persistence, and bioavailability, ultimately determining ecological exposure and impacts, while highlighting key research gaps for future environmental risk assessment.

Clay minerals, fine particles, organic matter, and Fe/Al/Mn oxides provide reactive interfaces that regulate sweetener sorption, mobility, and transformation. Their surface area, charge, mineralogy, interlayer spaces, and exchangeable cations determine interactions with polar and ionizable compounds through adsorption, interlayer retention, ion exchange, hydrogen bonding, van der Waals forces, and cation bridging [71,72]. Smectites, vermiculites, illites, kaolinites, and metal oxides differ substantially in these properties, while mineral–organic interfaces can additionally regulate water retention, diffusion, redox processes, microbial accessibility, and organic matter transformation [73,74]. Soil pH further affects sweetener speciation and surface charge, thereby influencing sorption, mobility, bioavailability, and biodegradation [75]. Under alkaline conditions, anionic species may become more mobile because of reduced sorption to negatively charged surfaces, although polyvalent cations can promote cation bridging. Biodegradation also depends on microbial activity, adaptation, rhizosphere effects, and contaminant accessibility, with saccharin and cyclamate generally being more biodegradable than sucralose, while acesulfame shows variable persistence [71,76,77,78,79].

Soil type and structure strongly influence transport. Sandy, low-organic-carbon soils generally favor mobility, whereas clay- and organic matter-rich soils enhance retention. However, macropores, cracks, root channels, and earthworm burrows can promote preferential flow, allowing highly soluble sweeteners to bypass reactive domains and reach deeper layers. Thus, strong microscale sorption does not necessarily prevent field-scale transport, and laboratory K_d_ values should be interpreted cautiously when extrapolated to structured soils. Redox gradients may further affect sweetener transformation and Fe/Mn oxide stability, particularly in waterlogged or wastewater- and sludge-amended soils.

The rhizosphere represents an additional hotspot of sweetener mobility, transformation, and bioavailability. Root exudates can alter pH, compete for sorption sites, complex metals, increase dissolved organic carbon, and stimulate microbial activity, potentially promoting either desorption and transport or biodegradation. Enhanced mobility may also facilitate plant uptake and soil-to-plant transfer, particularly in agricultural systems receiving reclaimed water or organic wastes [80], while soil bioavailability may influence biological responses in soil organisms such as *Folsomia candida* [46]. Overall, sweetener fate reflects interactions among chemical properties, soil mineralogy, organic matter, pH, structure, redox conditions, moisture, and microbial and rhizosphere processes. However, systematic comparisons among soil types remain scarce, limiting predictions of persistence, mobility, biodegradation, and transformation product formation and highlighting the need for environmentally realistic, soil-specific investigations.

#### 3.2.2. Persistence, Mobility, Degradation, and Environmental Transport of Sweeteners

Soil is a fundamental component of terrestrial ecosystems, supporting plant growth and diverse microbial and faunal communities while linking the lithosphere, hydrosphere, atmosphere, and biosphere through nutrient cycling, organic matter decomposition, water filtration, and carbon storage [81,82,83,84]. Its heterogeneous physicochemical properties, including pH, moisture, temperature, redox conditions, organic matter, and mineral composition, strongly regulate contaminant mobility, persistence, bioavailability, and transformation [85,86,87,88,89,90]. Artificial sweeteners have increasingly attracted attention as emerging soil contaminants because of their widespread use and continuous release through reclaimed water irrigation, sewage sludge and biosolid application, manure, landfill leachates, industrial discharges, and atmospheric deposition [91,92,93]. Once introduced, they may undergo sorption, biodegradation, leaching, plant uptake, and transformation depending on the compound properties and local environmental conditions [94,95,96].

Artificial sweeteners exhibit substantially different environmental fates. Sucralose is generally more persistent than saccharin and cyclamate, which undergo more rapid microbial transformation, whereas acesulfame-K shows intermediate or variable behaviour [97,98,99]. Sucralose has also been proposed as a conservative tracer of anthropogenic contamination because of its persistence and mobility in aquatic systems [100,101,102,103,104]. However, its behaviour in soils appears to be more complex, with low or undetectable concentrations frequently reported in agricultural soils [61,105], highlighting the importance of soil-specific processes. The sorption and mobility of anionic sweeteners such as acesulfame and saccharin are influenced by pH, ionic strength, cation composition, and organic carbon content, with increasing pH potentially reducing sorption and favoring mobility and groundwater transport [106]. Persistence does not necessarily imply biological inactivity: sucralose and acesulfame-K have been associated with changes in microbial diversity, enzymatic activity, and nutrient cycling in terrestrial and wetland systems [48,107,108].

Interactions with plants, soil organisms, and co-occurring contaminants further complicate sweetener behaviour. Aspartame and saccharin can be taken up by wheat roots and translocated to aerial tissues, where biotransformation generates metabolites potentially more ecotoxic than the parent compounds, indicating a possible pathway for soil-to-plant transfer and secondary contaminant formation [68]. Sweeteners also commonly co-occur with pharmaceuticals, antibiotics, pesticides, and metals, which may alter their bioavailability, degradation, and biological effects. For example, mixtures of pharmaceuticals and sweeteners have been associated with oxidative stress, membrane damage, and pigment reduction in wheat and barley [60,109]. Overall, sweetener fate in terrestrial environments reflects interactions among compound-specific properties, soil characteristics, microbial and plant processes, and co-occurring contaminants. Nevertheless, important uncertainties remain regarding long-term accumulation, transformation products, chronic low-dose exposure, mixture effects, and multigenerational responses. Integrated approaches combining soil chemistry, microbiology, ecotoxicology, and environmental modeling are therefore needed to improve risk assessment of sweeteners as emerging terrestrial contaminants.

## 4. Discussion

### 4.1. Sweetener-Provoked Oxidative and Genomic Stress Mitigation in Soils

The genotoxicity of artificial sweeteners remains poorly understood, and a clearer understanding is essential to safeguard genomic integrity and homeostasis not only in soil organisms but also in all participants in the food chain. Although studies on the genotoxicity of artificial sweeteners such as aspartame, saccharin, and sucralose in animals have produced conflicting results, their potential genotoxic effects on plants remain largely unexplored. This issue is particularly important because plant health and productivity are directly associated with soil fertility and ecosystem stability [110,111,112,113,114,115].

Under natural environmental conditions, detecting sucralose-induced genotoxicity in plants is highly challenging due to the complexity of soil matrices and the influence of multiple environmental factors. Therefore, additional in vitro and in vivo studies using plant cells and model plant organisms are required to determine whether sucralose can induce genetic mutations, chromosomal aberrations, DNA damage, or other genomic alterations. Given the currently available evidence suggesting both direct and indirect interactions between artificial sweeteners and genetic material across various organisms, further investigations are necessary to clarify the genotoxic potential of sucralose, particularly when combined with other food additives or environmental contaminants. Such studies are especially relevant within the broader context of the environmental genotoxicity associated with artificial sweeteners.

In addition to direct effects on plants, artificial sweeteners may also influence soil microbiota, which plays a fundamental role in nutrient cycling, humus formation, organic matter decomposition, and maintenance of soil fertility. Soil microorganisms, including bacteria, fungi, and actinomycetes, are directly involved in biochemical processes responsible for the stabilization of soil organic matter and the synthesis of humic substances. Disturbances in microbial communities caused by the long-term accumulation of artificial sweeteners may therefore negatively affect soil biochemical balance and ecosystem functioning.

Changes in microbial diversity and activity may alter key soil parameters, including pH, enzymatic activity, nutrient availability, and carbon turnover. Since many artificial sweeteners are highly persistent and resistant to biodegradation, their continuous introduction into the environment through wastewater, sewage sludge, irrigation systems, or landfill leachates may lead to chronic exposure of soil ecosystems. In the long term, elevated concentrations of artificial sweeteners may contribute to alterations in soil fertility, humus synthesis, and microbial homeostasis, emphasizing the importance of investigating these environmental effects in greater detail.

Another important aspect of artificial sweeteners in soils is their potential to induce oxidative stress. Several studies suggest that some artificial sweeteners may stimulate the formation of reactive oxygen species (ROS), leading to oxidative damage in microorganisms, plants, and other soil organisms. Excessive ROS production may compromise membrane integrity and the stability of proteins, lipids, and nucleic acids, thereby disrupting normal cellular metabolism and physiological processes. In soils, oxidative stress may additionally interfere with microbial enzymatic systems responsible for organic matter degradation and nutrient cycling.

The persistence of oxidative stress conditions may also contribute to reduced microbial activity and impaired soil ecological functions. Consequently, future environmental studies should focus on evaluating oxidative stress biomarkers in soil organisms exposed to artificial sweeteners, as well as on determining the relationships among sweetener concentration, persistence, and oxidative damage under different soil conditions. Such investigations are essential for understanding the long-term ecological risks posed by the accumulation of artificial sweeteners in terrestrial ecosystems.

One possible strategy to mitigate these environmental impacts is replacing highly persistent artificial sweeteners with natural alternatives that are more biodegradable in soils. Compounds such as perillartine and neohesperidin dihydrochalcone have attracted attention as potential alternatives due to their natural origin and potentially lower environmental persistence. Furthermore, incorporating antioxidant compounds, including polyphenols, terpenoids, and carotenoids, may help reduce oxidative stress induced by artificial sweeteners by scavenging reactive oxygen species and protecting cellular structures from oxidative damage. The combined application of biodegradable sweeteners and natural antioxidant compounds may therefore represent a promising approach for reducing the environmental risks associated with sweetener contamination in soils.

### 4.2. Sweetener-Provoked Environmental Stress and Its Mitigation

The environmental burden associated with artificial sweeteners is increasing, driven by their persistence in ecosystems and their potential to transform into more harmful derivatives.

Robertson et al. [116] examined the long-term persistence of sucralose in groundwater by conducting three sampling snapshots of a well-constrained wastewater plume in Canada (the Long Point septic system) over a 6-year period from 2008 to 2014. A shrinking sucralose plume was observed in 2014, compared to earlier sampling; as sucralose use was likely increasing during this period, this observation provides clear evidence of degradation. However, depletion of sucralose from a mean of 40 μg/L in the proximal plume zone occurred at a relatively slow rate over a period of several months to several years. Furthermore, septic tank effluent and impacted groundwater at six other sites in Canada were examined, and it was found that sucralose was present in all septic tank effluent samples (6–98 μg/L, *n* = 32) and all groundwater samples (0.7–77 μg/L, *n* = 64). Even though sucralose degradation has been observed in the Long Point plume, its ubiquitous presence in the groundwater plumes at all seven sites suggests a relatively slow rate of decay in many groundwater septic plume environments. A similar conclusion regarding sucralose concentration in groundwater was reached in a more recent investigation conducted by Cavanaugh [117] in Miami-Dade County, Florida, US.

If current trends persist, environmental concentrations of sucralose and related compounds may eventually exceed critical thresholds, leading to significant oxidative and genotoxic effects across ecosystems. For this reason, there is an urgent need to develop strategies for the safe removal, reuse, and substitution of synthetic sweeteners with natural alternatives [118,119,120,121,122,123,124].

In this context, several natural sweeteners have been proposed as “green” alternatives to sucralose. These compounds are generally more bioavailable and readily biodegradable, and some also exhibit a higher sweetness index than sucralose. A comparative overview of their advantages and limitations is presented in Table 2.

All of the listed sweeteners are naturally derived and considered non-toxic, with several compounds—such as perillartine and neohesperidin—exhibiting sweetness intensities three to five times higher than aspartame, saccharin, and sucralose. Beyond their sweetening properties, certain members of this group also exhibit antioxidant activity, supporting their potential use in sucralose-based beverages to reduce oxidative stress. Accordingly, replacing sucralose with natural alternatives, or incorporating them in combination within food formulations, represents a viable and necessary approach to the development of more environmentally sustainable dietetic products [125,126,127].

Natural sweeteners have received increasing attention as alternatives to synthetic sweeteners due to their low caloric value and potential health benefits. Huang et al. comprehensively reviewed the physicochemical properties, biological activities, and extraction technologies of steviol glycosides from *Stevia rebaudiana* [128], highlighting their growing application in the food industry as natural sugar substitutes. Although the review primarily focuses on food science and industrial processing, the expanding consumption of steviol glycosides suggests that their environmental release is also likely to increase. Consequently, future studies should evaluate the occurrence, persistence, transport, transformation products, and ecotoxicological effects of these compounds in terrestrial ecosystems, as such information remains scarce compared with that on artificial sweeteners such as sucralose, saccharin, and acesulfame. Another natural sweetener to be evaluated in this regard is perillartine [129,130].

Natural sweeteners such as steviol glycosides, hesperetin derivatives, and perillartine have attracted increasing interest as potential alternatives to synthetic sweeteners due to their natural origin and, in some cases, greater biodegradability. However, the available evidence remains insufficient to conclude that their widespread substitution would reduce the overall environmental burden. Comparative ecotoxicological studies, environmental fate assessments, and life-cycle analyses are still scarce for most natural sweeteners. Therefore, replacing one class of sweeteners with another should be supported by comprehensive evaluations considering persistence, transformation products, ecotoxicity, resource consumption, and environmental emissions throughout their life cycle.

The growing concern regarding emerging contaminants in terrestrial ecosystems is not limited to artificial sweeteners. Like soil microplastics, sweeteners may interact with soil physicochemical properties, microbial communities, plants, and soil invertebrates, potentially altering key ecological processes. As highlighted in recent reviews on soil microplastics [131], understanding the environmental behavior of emerging contaminants requires an integrated assessment of their persistence, transport, bioavailability, and ecological effects under realistic environmental conditions. A comparable multidisciplinary approach should be adopted for artificial and natural sweeteners, whose occurrence and environmental fate in soils remain poorly understood despite their increasing consumption and continuous release into the environment.

Another important aspect that deserves attention is the potential synergistic interaction between sucralose and micro- and nanoplastics (MNPs), which has not yet been systematically investigated. Considering the increasing co-occurrence of these emerging contaminants in terrestrial environments, their combined presence may influence their environmental fate, transport, bioavailability, and ecotoxicological effects. This hypothesis is particularly relevant for MNPs associated with PFAS, since these highly fluorinated compounds share certain physicochemical characteristics with sucralose, including high environmental persistence and resistance to degradation [14,66,131]. Therefore, future studies should evaluate whether MNPs can act as vectors for artificial sweeteners, potentially modifying their mobility, bioaccumulation, and toxicity in soil organisms under environmentally realistic exposure scenarios.

### 4.3. Sweetener Concentration Monitoring in Soils

Reported environmental concentrations of sweeteners are considerably more numerous for aquatic matrices than for soils, although the available evidence is heterogeneous among compounds and geographical regions. Sucralose, acesulfame potassium, saccharin, and cyclamate have been detected in both water and soil matrices, whereas quantitative information on aspartame and particularly steviol glycosides in soils remains scarce. Reported concentrations also vary substantially depending on the environmental matrix, sampling location, physicochemical characteristics of the soil, hydrological conditions, and analytical methodology. Therefore, direct comparison among studies should be interpreted with caution.

Table 3 summarizes selected quantitative data reported for sucralose, aspartame, saccharin, acesulfame potassium, cyclamate, and steviol glycosides in soil and aquatic matrices. The reported concentrations illustrate the marked difference in data availability between terrestrial and aquatic environments. For example, several studies have reported concentrations of sucralose, acesulfame potassium, saccharin, and cyclamate in surface waters, whereas corresponding measurements in soils are available from substantially fewer locations and studies. In some cases, soil concentrations have not been reported or compounds were not detected, highlighting the difficulty of establishing representative environmental concentration ranges for terrestrial systems.

The available data should therefore be regarded as an initial quantitative framework rather than a comprehensive assessment of environmental exposure. Differences in sampling strategies, detection limits, soil properties, water chemistry, and analytical procedures may contribute to the variability among reported values. Nevertheless, the comparison provides useful evidence that the environmental occurrence of sweeteners cannot be adequately characterized from aquatic monitoring alone and supports the need for further soil-specific investigations. The environmental concentrations summarized here also provide a basis for considering how the chemical properties of individual sweeteners influence their distribution, persistence, and interactions with environmental matrices.

As for the determination of artificial sweeteners, spectrophotometric, chromatographic, and electrochemical approaches have all been employed for analytical purposes. Among these, chromatographic techniques remain the most widely used, sensitive, and reliable methods for sweetener detection. However, the vast majority of analytical protocols have been developed primarily for food and beverage matrices and, more recently, for products such as e-cigarettes, whereas comparatively little attention has been devoted to soil matrices. This represents a significant limitation, considering the increasing environmental relevance of artificial sweeteners as emerging soil contaminants [136,137,138].

Chromatographic methods, particularly those coupled with spectroscopic or mass spectrometric detection systems, offer excellent selectivity and sensitivity, enabling the quantification of sweeteners at nanomolar or even picomolar levels in complex environmental samples. Nevertheless, these techniques generally require sophisticated instrumentation, extensive sample preparation, high operational costs, and relatively long analysis times. Their direct application to soils is additionally complicated by the heterogeneous composition of soil matrices, including organic matter, minerals, and humic substances, which may interfere with extraction efficiency and analytical detection [139,140,141,142].

In contrast, direct electrochemical methods represent potentially faster, simpler, and more cost-effective alternatives for sweetener determination. Despite these advantages, their practical application remains limited, largely due to challenges associated with electrode modification, signal overlap, and insufficient peak separation. In soil samples, these limitations are even more pronounced because additional extraction and cleanup procedures are typically required before analysis. Consequently, the development of optimized electrochemical sensors and their integration with chromatographic systems such as HPLC may represent a promising direction for future environmental monitoring strategies [143,144,145].

Future analytical developments should focus on improving both the sensitivity and applicability of methods for detecting sweeteners in complex soil matrices. Greater attention should be given to electrochemical techniques, which remain underexplored for compounds such as sucralose despite their potential advantages in terms of simplicity, rapid analysis, portability, and cost-effectiveness. Further reductions in detection limits toward the picomolar range would facilitate the quantification of trace concentrations in agricultural soils and groundwater. Moreover, the integration of complementary analytical techniques, such as electrochemical sensors combined with chromatographic and mass spectrometric methods, may improve analytical reliability by enabling both rapid screening and accurate confirmation of target compounds and their transformation products. Future method validation should also systematically evaluate detection and quantification limits, matrix recovery rates, selectivity, reproducibility, and overall analytical costs to facilitate routine environmental monitoring.

Consequently, future studies on sweetener determination in soils should focus not only on improving the sensitivity, selectivity, and cost-efficiency of HPLC methods but also on developing portable, rapid, simple, and accurate electrochemical techniques suitable for environmental monitoring. In addition, indirect assessment of sweetener contamination in soils by quantifying them in groundwater may represent an alternative and complementary approach, particularly in areas where sweeteners can migrate from soils into subsurface aquatic environments.

To close this discussion, it is important to emphasize that the environmental behavior of artificial sweeteners should be considered within an integrated soil–plant–water continuum rather than as isolated compartments. Their persistence, mobility, and potential for bioaccumulation mean that even low but continuous inputs may lead to long-term ecological consequences that are not immediately apparent from short-term studies. This reinforces the need for multidisciplinary approaches combining soil chemistry, ecotoxicology, microbiology, and environmental analytical chemistry to properly assess their real environmental footprint.

Ultimately, addressing the challenges associated with artificial sweeteners in soils requires a dual strategy: improving analytical capabilities for their detection and monitoring while simultaneously advancing safer, more sustainable alternatives. The convergence of these approaches—ranging from more efficient detection in complex soil matrices to the development and application of biodegradable sweeteners and protective antioxidant compounds—offers a realistic pathway toward mitigating long-term risks. In this context, future research should prioritize not only contamination assessment but also preventive design of sweetener systems aligned with soil health preservation and overall ecosystem resilience.

## 5. Conclusions

Artificial sweeteners have emerged as a relevant group of emerging contaminants in terrestrial environments, with increasing evidence confirming their presence in soils in different geographic regions and land use systems. Although originally considered environmentally inert due to their low toxicity and high water solubility, recent studies demonstrate that these compounds can persist in soils, undergo partial transformation, and interact with key biological and physicochemical processes. Their occurrence is closely linked to anthropogenic activities such as wastewater irrigation, application of sewage sludge, and agricultural reuse of reclaimed water.

Current evidence indicates that artificial sweeteners may influence soil microbial communities, enzymatic activity, and higher trophic levels, including soil invertebrates and plants. Observed effects include changes in microbial diversity, shifts in metabolic pathways, and potential alterations in nutrient cycling processes. In addition, uptake and biotransformation in plants suggest that these compounds can enter terrestrial food webs, raising concerns regarding long-term ecological consequences. However, the extent and mechanisms of these effects remain insufficiently understood, particularly under field conditions and long-term exposure scenarios.

An additional and promising approach may involve indirect assessment of soil contamination by monitoring artificial sweeteners in groundwater systems, particularly in shallow aquifers closely connected to surface soils. Such integrated approaches could provide valuable insights into contaminant transport pathways and accumulation dynamics across environmental compartments.

As a potential mitigation strategy, substituting artificial sweeteners with natural alternatives, such as steviol glycosides and other plant-derived sweetening compounds, may help reduce the environmental burden on soil ecosystems. Natural sweeteners are generally considered more readily biodegradable and less persistent in environmental matrices, which could limit their accumulation in soils and thereby reduce long-term exposure of soil biota to synthetic xenobiotics. Nevertheless, the environmental behavior of “natural” sweeteners is not yet fully understood, and their large-scale agricultural production may introduce additional pressures on land use and ecosystem services. Therefore, while substitution strategies may represent a promising approach to alleviate chemical stress in soils, they must be evaluated within a broader sustainability framework that considers production chains, degradation pathways, and potential unintended ecological consequences. Future research should therefore prioritize soil-specific characterization of environmental fate and transformation, chronic and mixture effects, bioavailability, and standardized analytical approaches incorporating sorption parameters, Kd, and relevant soil properties.

In this regard, while current research clearly establishes the environmental relevance of artificial sweeteners in soils, important knowledge gaps remain regarding their long-term fate, ecological impacts, and transformation processes. Addressing these gaps will require the development of standardized analytical methods, interdisciplinary approaches, and combined soil–water–atmosphere monitoring strategies.

## Figures and Tables

**Table 1:** Biological effects, environmental behavior, and soil-related impacts of artificial sweeteners and related compounds.

Study	System/Matrix	Compounds	Approach	Main Findings	Ecological Relevance
General overview [41,42,43]	Gut microbiota	Multiple ASs	Review/experimental evidence	Alters microbial composition, metabolism, and possible transgenerational effects	Basis for soil microbiome analogy
Functional analogy [44,45]	Gut vs. soil microbiomes	-	Conceptual comparison	Soil and gut microbiomes share functional roles in system stability	Supports soil-microbiome extrapolation
Environmental knowledge gap [44,45]	Soil ecosystems	ASs (general)	Review	Limited mechanistic data on soil biological effects	Major research gap identified
Lin et al. [46]	Soil fauna (*F. candida*)	SUC, SAC, CYC, ACE	Multi-omics	Reproductive stimulation at low doses; microbiome shifts; multi-generational effects	Dose-dependent ecological disruption
Lin [47]	Earthworm (*E. fetida*)	ACE	Multi-omics	Metabolic disruption; oxidative stress; microbiome alteration	Soil fauna toxicity at low concentrations
Marsh soil study [48]	Soil–water interface	SUC	Microbial ecology	Reduced microbial activity; shift toward cyanobacteria dominance	Aquatic–terrestrial ecosystem alteration
Zeng [49]	Engineered nitrification systems	SUC + TCS	ARG analysis	Enhanced antibiotic resistance gene dissemination	Risk of ARG spread via soil reuse systems
Fu [50]	Soil–water continuum	Cyclamate + antibiotics	ARG + microbial analysis	Increased ARG mobility and multidrug resistance enrichment	Non-antibiotic drivers of AMR
O’Brien [51]	Biosolids	Multiple ASs	Field monitoring	Accumulation in biosolids; limited ecological risk; soil input quantified	Land application exposure pathway
Yue [52]	Lake riparian soils	SUC, ACE, SAC, CYC	Spatial ecology	Sediment accumulation; sucralose persistence; transformation hotspots	Long-term sediment reservoir behavior
A. cepa studies [53,54,55,56]	Plant bioassays	SUC, SAC, AS mixtures	Genotoxicity tests	Chromosomal damage and micronuclei formation	Evidence of plant-level genotoxicity
A. cepa mixture study [57]	Plant bioassays	SUC + mixtures	Ecotoxicology	Increased genotoxicity in mixtures vs. single compounds	Mixture toxicity amplification
Genotoxic precursors [58,59]	Environmental organisms	Cyclamate metabolites, SUC intermediates	Toxicological evidence	Potential DNA-level effects are suggested but poorly characterized	Knowledge gap in soil genotoxicity
Li [60]	Wheat (*T. aestivum*)	ASP, SAC	Plant uptake	Uptake + phase II metabolism (methylation, glucuronidation)	First evidence of plant biotransformation
Elveren [61]	Wheat and barley	AS + pharmaceuticals	Physiological assays	Oxidative stress, pigment reduction, enzyme activation	Mixture-induced plant stress
Costello et al. [62]	Three North American prairie species	Sucralose	Plant ecotoxicology; physiological and phenotypic endpoints	Environmentally relevant concentrations of dissolved sucralose affected gas exchange, chlorophyll content, and flowering hue	Demonstrates that environmentally relevant SUC exposure may affect plant physiology and reproductive/phenotypic traits, providing evidence for potential phytotoxicity in terrestrial ecosystems
Qureshi et al. [63]	*Allium cepa* root tip cells	Natural and artificial sweeteners	Phyto-genotoxicity assay	Sweetener exposure induced cytogenetic alterations in *A. cepa* root tip cells, demonstrating potential genotoxic effects	Supports the use of plant cytogenetic models for evaluating the genotoxicity of sweeteners and highlights possible risks to plants exposed to contaminated soils
Thallmann [64]	Agricultural soil	AS + OMPs	Sorption experiments	Strong dependency on pH, ionic strength, soil OC	Mobility and transport variability

**Table 2:** Natural sweeteners as potential alternatives to sucralose.

Sweetener	Natural Source	Relative Sweetness	Reference
Steviol glycosides	*Stevia rebaudiana*	~195× sweeter than sucrose	[118]
Glycyrrhizin	*Glycyrrhiza glabra*	30–50×	[119]
Perillartine	*Perilla frutescens*	~2000×	[120]
Tagatose	Dairy-derived (e.g., lactose)	0.75× (slightly less sweet)	[121]
Carob gum + tannin adducts	*Ceratonia siliqua*, *Prosopis glandulosa*	0.75×	[122]
Miraculin	*Synsepalum dulcificum*	Not sweet itself; modifies sour taste to sweet	[123]
Neohesperidin dihydrochalcone	Citrus-derived flavonoid	~3000×	[124]

**Table 3:** Reported concentrations and detection of selected sweeteners in soil and aquatic matrices.

Sweetener	Matrix	Location	Reported Concentration	Detection/Frequency	Reference
Sucralose	Soil	China	0.18 μg/kg	Detected	[132]
Sucralose	Soil	Various	0.16–35 μg/kg	Detected	[133]
Sucralose	Surface water	Various	0.0001–600 μg/L	Variable	[133]
Acesulfame-K	Soil	China	0.04 μg/kg	Detected	[132]
Acesulfame-K	Surface water	China	0.22 μg/L	Detected	[132]
Saccharin	Soil	China	0.06 μg/kg	Detected	[132]
Saccharin	Surface water	China	0.06 μg/L	Detected	[132]
Cyclamate	Soil	China	0.06 μg/kg	Detected	[132]
Cyclamate	Surface water	China	0.08 μg/L	Detected	[132]
Aspartame	Soil	China	ND	0/98 samples	[134]
Aspartame	Surface water	China	≤0.21 μg/L	Detected	[134]
Steviol glycosides	Soil	—	NR	Environmental soil data scarce	—
Stevioside	Wastewater influent	Serbia	0.37 μg/L	Detected in influent; 100% removal	[135]

## Data Availability

No new data were created or analyzed in this study. Data sharing is not applicable to this article.

## Appendix A

This appendix provides additional methodological information supporting the literature search, screening, and selection procedures described in the main text. Its purpose is to improve the transparency and reproducibility of the review by documenting the database-specific search strategies and summarizing the study selection process. It includes search queries and relevant database-specific parameters, clarifying how the literature was retrieved. Second, the study selection process is summarized, including the allocation of studies to the present review and the companion review.

### Appendix A.1. Database-Specific Search Strategies

The literature included in this review was selected through a structured literature search and screening process designed to identify relevant publications addressing the occurrence, environmental fate, and potential biological effects of natural and artificial sweeteners in soils and at the soil–water interface. The search focused on the presence of sweeteners in terrestrial environmental matrices, their persistence and mobility, and their potential ecotoxicological effects, including genotoxicity and oxidative stress. The databases consulted were Web of Science, Scopus, and PubMed.

The search strategy combined terms describing sweeteners and sugar substitutes with terms related to environmental matrices and, where appropriate, their potential biological effects. Wildcard characters and Boolean operators were used to account for variations in terminology and to combine the principal concepts relevant to the review. The following search strings illustrate the structure of the queries used to identify publications concerning sweeteners in terrestrial environmental matrices:

(Sweeteners OR Sugar Substitutes OR Edulcorants OR Sucralose OR Aspartame OR Saccharin OR Acesulfame K OR Cyclamate OR Steviol Glycosides)

AND

(Soil OR Sediment OR Lithosphere OR Terrestrial Ecosystems OR Wetland OR Marsh Soil-Water Interface OR Groundwater)

AND

(Detection OR Environmental Fate OR Environmental Impact OR Degradation OR Decomposition OR Biodegradation OR Metabolism)

The search strings did not always reproduce the review keywords verbatim; they also incorporated semantically related terms from the same semantic fields to account for terminological variation across the literature.

The databases were consulted on seven occasions: on 12th and 26th of June; 10th, 24th, and 31st of July; and 14th and 25th of August 2026. The literature search covered publications from 2006 to the first half of 2026. In addition to database searching, reference lists and citation links were examined to identify further publications potentially relevant to the objectives of the review. This supplementary citation-tracking procedure was used to broaden coverage of the literature and identify relevant studies that might not have been retrieved by the initial queries.

### Appendix A.2. Literature Screening and Study Selection

Study selection proceeded through an initial screening of titles and abstracts, followed by examination of the full texts of potentially relevant publications. Inclusion was based primarily on relevance to the scope of the review, particularly the occurrence and environmental fate of sweeteners in soil systems and at the soil–water interface, along with their potential biological and ecotoxicological effects. The methodological characteristics and scientific contribution of each study were also considered during the assessment of its relevance. Preprints and short conference papers without a completed peer-review process were excluded from the final evidence base, as were theses and publications considered outside the scope of the review.

The initial search and subsequent screening identified 210 sources in total. Following the removal of duplicate records, preprints, theses, and studies deemed irrelevant to the scope of the research, 98 studies were retained for further consideration. Of these, 26 were allocated to a companion review focusing on the analytical determination of sweeteners in soil matrices. The remaining 72 articles constitute the evidence base of the present review, which focuses on the occurrence, environmental fate, and potential biological effects of sweeteners in terrestrial systems and at the soil–water interface. Data regarding the literature screening can be seen in Table A1.

**Table A1:** Information on Literature Screening for the Review.

Date of the Search Query	Scopus	WoS	PubMed	Number of Studies Analyzed	Number of Studies Excluded (Moved to the Companion Review)
12 June 2026	15	10	12	37	23 (4)
26 June 2026	12	12	15	39	27 (5)
10 July 2026	9	8	9	26	18 (3)
24 July 2026	7	6	7	20	12 (3)
31 July 2026	8	6	5	19	10 (1)
14 August 2026	12	18	8	38	28 (5)
25 August 2026	12	12	7	31	20 (5)
Total	75	72	63	210	138 (26)

This selection process was intended to establish a relevant and sufficiently broad evidence base for assessing the current state of knowledge, identifying limitations in the available evidence, and highlighting priorities for future research. The search strategy and selection process should be interpreted in accordance with the narrative scope of the review.
